# Contributions of Chondroitin Sulfate, Keratan Sulfate and N-linked Oligosaccharides to Inhibition of Neurite Outgrowth by Aggrecan

**DOI:** 10.3390/biology9020029

**Published:** 2020-02-12

**Authors:** Thomas M. Hering, Justin A. Beller, Christopher M. Calulot, Diane M. Snow

**Affiliations:** 1 Spinal Cord and Brain Injury Research Center, University of Kentucky, Lexington, KY 40536, USA; jabeller@gmail.com (J.A.B.); chris.calulot@uky.edu (C.M.C.); diane.snow@tcu.edu (D.M.S.); 2 Department of Biomedical Engineering, Case Western Reserve University, Cleveland, OH 44106, USA; 3 Department of Biology, Texas Christian University, Fort Worth, TX 76129, USA; 4 Department of Anatomy and Neurobiology, University of Kentucky, Lexington, KY 40536, USA

**Keywords:** neurite outgrowth, aggrecan, chondroitin sulfate, keratan sulfate, N-linked oligosaccharides

## Abstract

The role of proteoglycans in the central nervous system (CNS) is a rapidly evolving field and has major implications in the field of CNS injury. Chondroitin sulfate proteoglycans (CSPGs) increase in abundance following damage to the spinal cord and inhibit neurite outgrowth. Major advances in understanding the interaction between outgrowing neurites and CSPGs has created a need for more robust and quantitative analyses to further our understanding of this interaction. We report the use of a high-throughput assay to determine the effect of various post-translational modifications of aggrecan upon neurite outgrowth from NS-1 cells (a PC12 cell line derivative). Aggrecan contains chondroitin sulfate, keratan sulfate, and N-linked oligosaccharides (N-glycans), each susceptible to removal through different enzymatic digestions. Using a sequential digestion approach, we found that chondroitin sulfate and N-glycans, but not keratan sulfate, contribute to inhibition of neurite outgrowth by substrate-bound aggrecan. For the first time, we have shown that N-linked oligosaccharides on aggrecan contribute to its inhibition of neuritogenesis.

## 1. Introduction

Proteoglycans (PGs) are a class of complex macromolecules commonly associated with the cell surface and the extracellular matrix. PGs are comprised of a core protein that is modified post-translationally by attachment of a variety of glycosaminoglycans (GAGs) and N-linked oligosaccharides. Depending on the specific sugars that make up the GAG chains, they are separated into four distinct groups. Chondroitin sulfate (CS), dermatan sulfate (DS), heparan sulfate (HS), and keratan sulfate (KS) differ in their carbohydrate composition and tend to cause different cellular responses in many physiological systems [1]. Of particular importance is the formation of a glial scar following spinal cord injury and the increased abundance of chondroitin sulfate proteoglycans (CSPGs) in these scars. The increased CSPG content of the glial scar is a major impediment to neuronal regeneration and it is believed that by overcoming this impediment, one can promote reconnection of the disconnected portions of the spinal cord. Much research in the role of proteoglycans in CNS physiology focuses on the CSPGs and their ability to inhibit neurite outgrowth. Numerous investigations have indicated that CSPGs inhibit outgrowth of neurons [2,3,4,5,6]. In some circumstances, however, CSPGs may stimulate neurite outgrowth [7,8].

**Figure 1:**
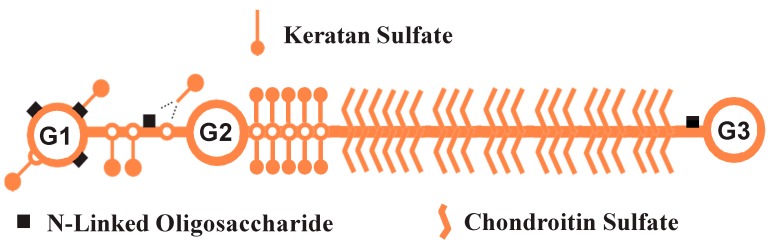
Schematic structure of bovine aggrecan. The aggrecan core protein comprises three globular domains (G1, G2 and G3) with an extended interglobular domain (IgD) between G1 and G2, and a larger extended region between G2 and G3. Keratan sulfate is substituted on the core protein primarily in a KS-rich region near G2. KS chains are also found on the G1 domain and IgD. Chondroitin sulfate chains are substituted throughout the extended region between the KS-rich region and G3. N-glycans are found on G1 and the IgD, and near G3.

There is a great deal of uncertainty concerning the specific components of the proteoglycan macromolecule that affect neurite outgrowth. Aggrecan is a complex “prototypical” PG used in many PG–neurite interaction experiments [2,4,9,10]. Aggrecan is one of the CSPGs of perineuronal nets (PNNs) [11,12] a layer of extracellular matrix appearing late in development, that surrounds soma, dendrites and initial axon segments. PNNs play a role in the control of CNS plasticity, and their removal can restore plasticity in the adult CNS. The interaction of PGs with cells can be divided into a hierarchy of possible interactions with its multiple components. Proteoglycans comprise a core protein substituted with GAG chains, and variably substituted with N-linked oligosaccharides. The primary sequence of the core protein itself represents the first level of possible PG interaction and the primary sequence of the GAG chains represents a second level of interaction. The sugar molecules of the GAG chains can be sulfated in various patterns, allowing for the construction of complex motifs and varieties of sulfated disaccharides leading to the third level of interaction. Proteoglycans can be substituted with N-linked oligosaccharides (N-glycans) at specific sites on the core protein. The structure of bovine aggrecan is shown in Figure 1. In this study we investigated interactions of GAG chains with neurons and also investigated a possible fourth level of interaction between neurites and N-glycans on the aggrecan core protein.

Advances in the field have made a significant impact on our understanding of how proteoglycans may interact with neurites. For instance, identification of receptors for CSPGs and HSPGs [13,14,15] afford us targets for pharmacological treatment, and advances in synthetic chemistry allow us to produce complex and specific carbohydrate polymers with which to test these interactions [16]. In addition to direct interactions between CS chains and cellular receptors, CS chains may competitively bind to neurotropins such as nerve growth factor (NGF). Since proteoglycan molecules are complex, it is possible that inhibition of neurite outgrowth may be due to multiple components of the PG. In this report we attempt to further answer this question by testing the inhibitory activity of aggrecan sequentially degraded by enzymes to remove specific carbohydrate moieties.

Bovine aggrecan (Figure 1), the well-characterized CSPG used in this study, contains over 100 sites for addition of chondroitin sulfate chains, 24 or more sites for addition of keratan sulfate chains, and up to 5 N-linked oligosaccharides [17]. The presence of these three post-translational modifications on aggrecan can make interpretation of many studies difficult. Considering the difficulty of interpreting contributions of different components of a single PG, it is even more difficult to interpret numerous published experiments using a mixture of substrate-bound CSPGs with unknown compositions [18,19,20] and CSPGs suspended in solution [21,22].

Using Neuroscreen-1™ (NS-1) cells, a PC12 cell line derivative, we examined the contribution of the various post-translational modifications of aggrecan upon neurite outgrowth. PC12 cells were originally derived from a pheochromocytoma of the rat adrenal medulla having an embryonic origin from the neural crest [23]. They can stop dividing and differentiate into neuron-like cells when treated with nerve growth factor (NGF), and in response to the presence of NGF these cells extend neurites. NGF is known to bind to at least two classes of receptors: the tropomyosin receptor kinase (TrkA) and the low-affinity NGF receptor (LNGFR/p75NTR). In this report we are investigating the effect of the various modifications of aggrecan upon stimulation of neuritogenesis of NS-1 cells by NGF.

Prior evidence suggests that the removal of CS chains from CSPGs in the CNS can improve neurite outgrowth. Experimentation in vitro and in vivo shows that removal of the CS carbohydrate chains, through the action of the bacterial enzyme chondroitinase ABC (cABC), greatly increases the amount of neurite outgrowth [3,5,24,25]. There is also evidence to suggest that dermatan sulfate (DS) [26] and KS [3,27,28] of some PGs may also exert an inhibitory influence on neurite outgrowth. However, the contribution of N-glycans to the inhibitory effect of proteoglycans has not been explored. We therefore investigated whether N-glycans might also have an effect on neurite outgrowth. In this study, successive enzymatic digestions targeting CS, KS, and N-glycans were performed to examine the contribution of each component. Sequentially, cABC was used to remove CS, dual digestion with keratanase II and endo-β-galactosidase was employed to remove KS, and Peptide: N-glycosidase F (PNGaseF) digestion was used to remove N-glycans. Therefore, our approach tested four different forms of aggrecan: intact aggrecan, CS-depleted aggrecan, CS/KS-depleted aggrecan, and CS/KS/N-glycan-depleted aggrecan.

The NS-1 cell assay has been previously described by our lab [29] and others [30] and has been shown to be useful to quantify robust to subtle changes in neurite outgrowth. There is an abundance of published research on the effects of PGs on neurite outgrowth using various qualitative or semi-quantitative methods which would not have been sufficient to quantify the subtle effects of the individual constituents of aggrecan. To our knowledge, our laboratory is the first to use the NS-1 assay to quantitatively assess changes in outgrowth of neurites in response to substrate bound, variably deglycosylated aggrecan.

## 2. Materials and Methods

### 2.1. Preparation of Aggrecan

Aggrecan was prepared from bovine articular cartilage as previously described [31,32]. Briefly, articular cartilage from 1–2 year old steers was dissected from metacarpal-phalangeal joints, and proteoglycans were extracted in ice-cold dissociative solubilization buffer (4 M guanidine-HCL, 0.15 M sodium acetate, 50 mm EDTA, pH 6.3, with protease inhibitors). The cartilage tissue was extracted at 5 °C for 48 h, filtered, and then dialyzed at 5 °C for 16 h in 20 volumes of associative buffer (0.15 sodium acetate, 5 mM EDTA, pH 6.3 with protease inhibitors). Aggrecan was then purified through three separate equilibrium density gradient centrifugal separations. The first separation was carried out under associative conditions, wherein the sample was placed in a 2.5 M CsCl solution and centrifuged for 48 h at 5 °C at 40,000 rpm. The gradient resolved into three equal fractions called A1-A3, according to previous characterization. Fraction A1 (bottom 1/3 of associative gradient) was isolated and two volumes of dissociative buffer (5.5 M guanidine HCl, 5mM EDTA, 0.15 M sodium acetate, pH 6.3, with protease inhibitors) were added. The solution was stirred for 4 h and subsequently dialyzed overnight against 20 volumes of associative buffer. The second separation in associative buffer was performed in 3.5 M CsCl at 5 °C for 48 h at 40,000 rpm. The resulting bottom third of the sample (Fraction A1A1) was subjected to a final equilibrium gradient centrifugal separation under dissociative conditions. The gradient was cut into three equal fractions, called D1-D3, and the bottom fraction D1 was dialyzed against 0.1 M sodium acetate/5 mm EDTA, 0.05 M sodium acetate/2.5 mm EDTA, followed by three changes of water. The sample was lyophilized and stored at −80 °C.

### 2.2. Enzyme Digestions

Three enzymes were used to remove the different forms of oligosaccharides associated with aggrecan: Chondroitinase ABC (Sigma-Aldrich, St. Louis, MO; Lot 081M4093V and 042M4117V) was used to digest chondroitin sulfate chains from the aggrecan core protein. Endo-β-galactosidase (Sigma-Aldrich, St. Louis, MO; Lot 090M1909 and SLBC2405V) and keratanase II (Seikagaku, Tokyo, Japan; Lot E10122) were used in conjunction to remove keratan sulfate chains from the aggrecan core protein. Peptide N-glycosidase F (QA Bio, Palm Desert, CA; Lot 107.2A) was used to digest the N-linked oligosaccharides. Chondroitinase ABC digestion was carried out in a buffer of 50mM Tris and 60mM sodium acetate titrated with HCl to a pH of 8.0. The endo-β-galactosidase and keratanase II dual digestion was performed in a 30 mM sodium acetate buffer titrated with acetic acid to a pH of 5.9. For the Peptide N-glycosidase F (PNGase) digestion, the enzymatic reaction was performed in 100 mM Phosphate Buffered Saline (PBS) with a pH of 7.5.

Following adsorption of the aggrecan to the bottom of the wells of a collagen-coated 96-well plate and subsequent rinsing, the bound aggrecan was subjected to enzymatic digestion. We used a stepwise approach to remove the oligosaccharides from the aggrecan core protein. The first step was the removal of chondroitin sulfate chains using chondroitinase ABC. A total of 150 µL of solution was added to each well containing 0.25 U of chondroitinase ABC in the chondroitinase buffer and allowed to incubate for 2 h at 37 °C. Following digestion, cells were rinsed 3 times in 0.1 M PBS and either kept in 0.1 M PBS (CS-depleted aggrecan) or further incubated with 0.375 U of endo-ß-galacotosidase and 0.5 µU of keratanase II for 2 h at 37 °C. Following incubation, wells were rinsed with 0.1 M PBS, either remaining in 0.1 M PBS (CS-depleted and CS/KS-depleted) or further digested with 0.125 U of PNGase F for 2 h. Following PNGase F digestion wells were rinsed in 0.1 M PBS (CS/KS/N-glycan-depleted). NS-1 cells were then seeded at a density of 2000 cells/well. We determined that adsorbed enzymes had no non-specific effects upon neurite outgrowth (not shown) by performing the analysis using the same enzyme treatments in wells containing no aggrecan.

### 2.3. ELISA Assay

Wells of a collagen-coated 96-well plate were coated with and without aggrecan at 150 and 300 µg/mL. Subsets of these wells were then subjected to the three enzymatic digestions as described above. Following the enzymatic digestion, the wells of the 96-well plate were washed three times in 0.1 M PBS with 0.01% Tween-20 (PBS-T). Wells were then incubated in a mouse monoclonal antibody against the G1-domain of aggrecan (AbD Serotec, Celtic Molecular Diagnostics Ltd.; Mowbray, South Africa) at a dilution of 1:1000 in 10 mL of Ab diluent (3% bovine serum albumin, 0.005% Tween-20 in 0.1 M PBS) overnight at 4 °C. Wells were than washed with PBS-T in triplicate, followed by incubation with an anti-mouse IgG horseradish peroxidase-conjugated secondary antibody (Sigma-Aldrich; St. Louis, MO) at a dilution of 1:10,000 in Ab diluent for 2 h at 27 °C. Wells were then washed 3X with PBS-T, and incubated in 200 µL/well of SigmaFast OPD (Sigma-Aldrich; St. Louis, MO) for 30 min at 27 °C. Absorbance was then read at 450 nm.

### 2.4. Neurite Outgrowth Assay

The neurite outgrowth assays were performed as described in detail previously [29,33]. Briefly, collagen-coated 96-well plates were incubated with varying concentrations of aggrecan in 0.1 M phosphate buffered saline (PBS) overnight at 4 °C. Following adsorption of aggrecan, wells were left untreated or were treated with enzymes as described above. Subsequently, NS-1 cells (Cellomics, Inc.; Pittsburgh, PA; Neuroscreen-1 (Cellosaurus RRID:CVCL_JY59)) were seeded at a density of 2000 cells/well and allowed to attach and settle overnight. Varying concentrations of NGF diluted in NS-1 cell media (RPMI1640 supplemented with 10% horse serum, 5% FCS, and 1x penicillin/streptomycin) were added to the wells and NS-1 cells were cultured to permit neurite extension for 72 h. Cells were then fixed and visualized by immunofluorescence using a proprietary antibody, with a fluorescein isothiocyanate (FITC)-tagged secondary antibody, and Hoechst dye to visualize nuclei. The proprietary antibody used in our study was included in a Cellomics Neurite Outgrowth Kit obtained through Thermo Fisher Scientific (Catalog number K07-0001-1, Waltham, MA, USA). The kit included a primary antibody specific for neuron cell bodies and neurites. The antibody was diluted using 13.75 μL of Neurite Outgrowth Primary Antibody to 11 mL of 1X Neurite Outgrowth Buffer (provided with the kit).

### 2.5. Image Acquisition

Using a microscope with a motorized stage, 9 images from each well were captured at 200X magnification. Both the Hoechst and FITC channels were acquired sequentially. Images were then analyzed using the NeuriteTracer macro for ImageJ (NIH; Bethesda, MD, USA) to determine neurite length [34]. NeuriteTracer skeletonizes neurites and measures the total length of neurites in a given image. Nuclei were counted by NeuriteTracer, and verified by hand for comparison to automated values, and the neurite length per cell was calculated for each image. The automated analysis performed through NeuriteTracer calculates neurite length (in pixels)/cell. These values are converted to micrometers through a simple calculation. In this project, an Axiocam MRm (Carl Ziess Microscopy GmbH; Jena, Germany) was used for image acquisition with no binning. The physical size of a pixel on this camera is 6.45 μm × 6.45 μm. Using 200× magnification and 1x1 binning, the conversion factor was calculated as follows: Pixel Size = 6450 nm × 1/(200) = 32.25 nm/pixel or 0.03225 μm/pixel. For simplicity, neurite length is expressed in μm in this report.

### 2.6. Data Analysis

All analyses was conducted using GraphPad Prism 5 for Mac OS X (GraphPad Software; La Jolla, CA, USA). Neurite length was divided by their associated cell number and imported into GraphPad Prism. These values were then multiplied by the conversion factor (above) and expressed as μm/cell. The data tables were set up to express each neurite length as a factor of the log of the NGF concentration. This data was then used to fit a sigmoidal dose-response curve to each set of data. During this analysis, we also used GraphPad’s algorithm for determining the presence of any outliers (ROUT analysis) and they were removed from further analyses.

From the dose-response curves regressed through Prism, the maximal response was directly calculated. For determining the inhibitory activity of aggrecan, a separate data table for xy plot was constructed. The neurite lengths at a given concentration of NGF (y (dependent) variable) were plotted as a factor of the log concentration of aggrecan (x (independent) variable). In all cases, this produced a linear relationship. Subsequently, a line was fit to this data using linear regression. The slope of this line was considered the inhibitory activity.

In order to calculate the descriptive statistics (i.e., means), bar graph data tables were constructed for each concentration of NGF. Each treatment of aggrecan was plotted as its own group (x-variable). Neurite outgrowth values for that particular treatment were pasted into the associated column. Using GraphPad Prism, means, standard deviations, and standard errors were calculated. These data tables were further used to conduct statistical analysis. All statistical analysis for the descriptive statistics included a preliminary ANOVA followed by two post-hoc Dunnet analyses, one comparing each group to the no aggrecan control condition, and another comparing each group to the intact aggrecan condition. P-values to accept or reject the null hypothesis were set at an α value of 0.05.

## 3. Results

### 3.1. NS-1 Cells Extend Neurites in a Dose-Dependent Fashion When Exposed to Varying Amounts of NGF

**Figure 2:**
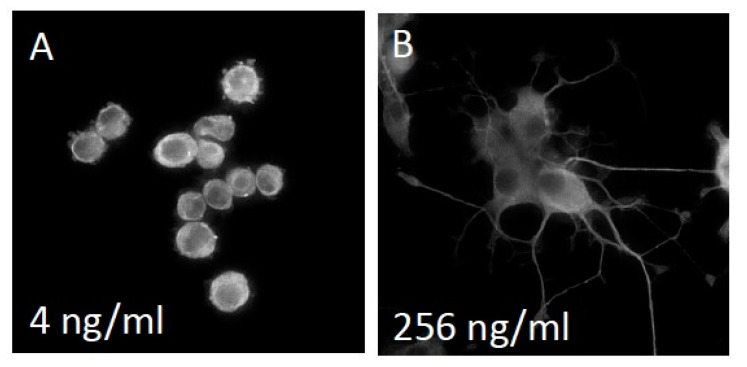
Neuroscreen-1™ (NS-1) cells extend neurites in a nerve growth factor (NGF) dependent manner. Representative micrographs of NS-1 cells grown on a collagen-coated surface following 72 h exposure to 0 and 256 ng/ml NGF are shown in panels (**A**) and (**B**), respectively.

The first series of experiments was to empirically determine an effective range of NGF concentrations over which to conduct neurite outgrowth experiments. We exposed NS-1 cells to varying concentrations of NGF for 72 h and analyzed neurite outgrowth as explained in the methods section. Figure 2 shows representative micrographs of NS-1 cells grown on a collagen-coated surface following 72 h exposure to 4 and 256 ng/mL NGF. In a pilot experiment, NS-1 cells were exposed to concentrations of NGF ranging from 0 ng/mL to 1000 ng/mL. An observable saturation of outgrowth was observed at 250 ng/mL (not shown). Subsequent experiments were usually conducted using 0.25 ng/mL, 31.25 ng/mL, 125 ng/mL, and 250 ng/mL NGF. The curve of neurite outgrowth vs. log [NGF] followed the predicted and typical sigmoidal dose-response curve.

### 3.2. ELISA Assay Confirms Adsorption to Wells and Reveals Greater Access to G1-Domain Following Enzymatic Digestion

**Figure 3:**
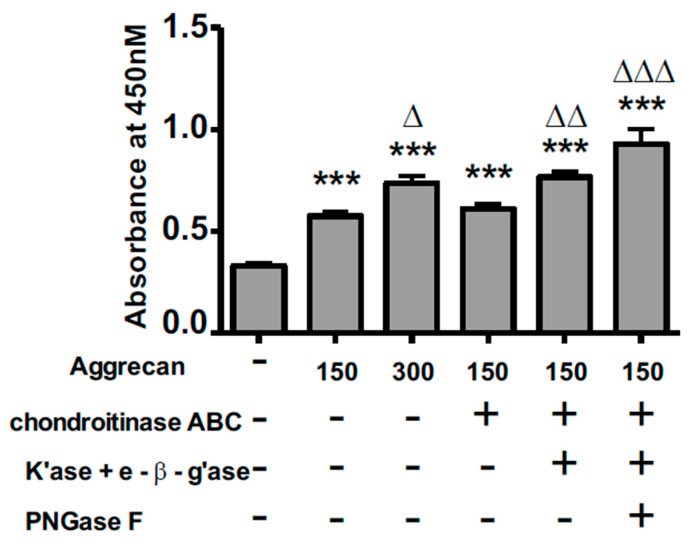
ELISA assay demonstrating aggrecan adsorption to wells. No aggrecan or aggrecan at 150 or 300 µg/mL was plated into wells of a 96-well plate and were subjected to the enzyme digestion protocol. Wells were reacted with primary antibody against the G1-domain, reacted with a horseradish peroxidase secondary antibody, incubated with peroxidase substrate, and the absorbance was read at 450 nm. ANOVA analysis was conducted followed by a Bonferroni post-hoc analysis. Asterisks (*) represent differences in absorbance from the no aggrecan condition while Δ represents differences from 150 µg/mL intact aggrecan. *** *p* < 0.001, Δ *p* < 0.05, ΔΔ *p* < 0.01, ΔΔΔ *p* < 0.001.

An ELISA assay was conducted to ensure adsorption of aggrecan to the surface of the collagen-coated wells. An antibody specific for the G1 domain of aggrecan was used. Figure 3 shows the results of the ELISA assay. As was expected, there was a significant increase in the absorbance of wells treated with aggrecan versus wells not treated with aggrecan. In addition, wells treated with 300 μg/mL aggrecan had a significantly higher absorbance, indicating that an aggrecan solution at 150 μg/mL did not saturate the surface. Surprisingly, we saw a significant increase in the absorbance of the CS/KS-depleted and CS/KS/N-glycan-depleted aggrecan compared to intact aggrecan, but no difference between the intact and CS-depleted aggrecan. The likely reason for this is due to the location of KS and N-glycans on the aggrecan core protein. KS chains are found in proximity to the G1–G2 region (Figure 1) and the majority of N-glycans are located on the G1 domain. Therefore, from this ELISA, we can conclude that aggrecan is adsorbing to the wells in a dose-dependent manner. Furthermore, confirming successful removal of carbohydrate, enzymatic digestions to remove KS and N-glycans increases antibody access to the G1 domain.

### 3.3. NS-1 Cell Neurite Outgrowth is Inhibited in a Concentration-Dependent Manner by Bound Aggrecan

**Figure 4:**
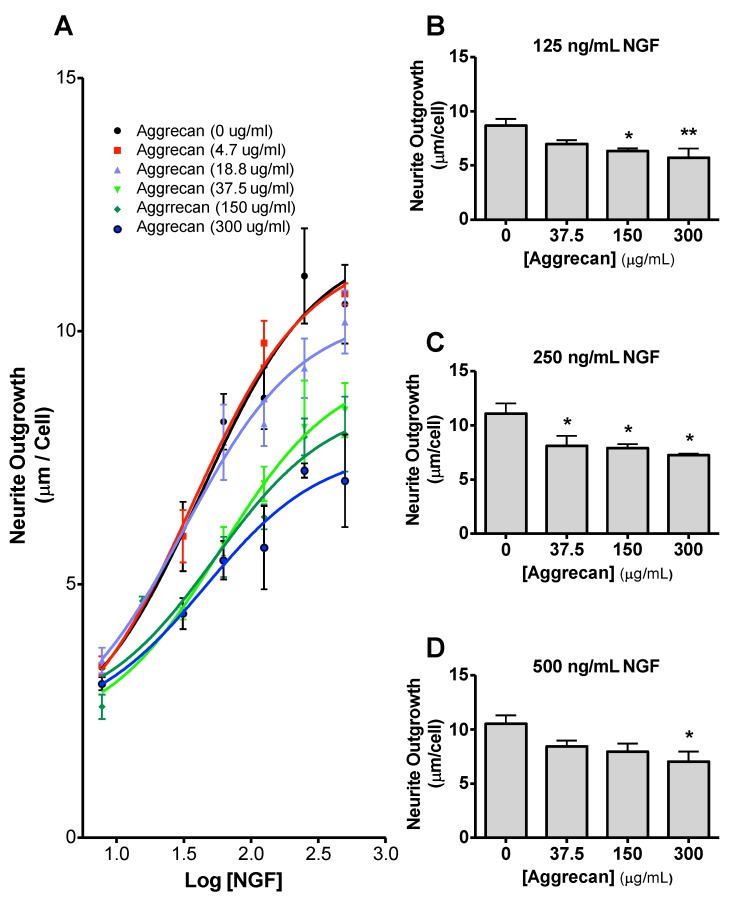
NS-1 neurite outgrowth is inhibited by aggrecan in a dose-dependent manner. Dose response curve for all concentrations of aggrecan analyzed (**A**). Neurite outgrowth at 125 ng/mL (**B**), 250 ng/mL (**C**) and 500 ng/mL (**D**) NGF in the presence of 0, 37.5, 150, or 300 µg.mL aggrecan. * *p* < 0.05, ** *p* < 0.01, compared to 0 µg/mL condition (Bonferonni post-hoc analysis).

Aggrecan was bound to the substrate using coating solutions of 0, 4.7, 18.8, 37.5, 150, and 300 µg/mL. Inhibition of neurite outgrowth was then assessed as described in the methods section. In this experiment, NS-1 cells were differentiated using a range of concentrations of NGF (0, 7.8, 15.6, 31.25, 62.5, 125, 250, and 500 ng/mL) and incubated to allow neurite outgrowth for 72 h. The NS-1 cells were then fixed, immunoreacted, and analyzed for total neurite length. There was an aggrecan concentration-dependent reduction in neurite outgrowth (Figure 4A). At 125 ng/mL μg/mL NGF (log [NGF] = 2.10) neurite outgrowth at aggrecan concentrations of 150 μg/mL (6.33 ± 0.24 μm/cell; *p* < 0.05), and 300 µg/mL (5.72 ± 0.82 μm/cell; *p* < 0.01) were significantly reduced (Figure 4B) when compared to NS-1 cells grown on the absence of substrate-bound aggrecan (8.68 ± 0.61 μm/cell). At 250 ng/mL NGF (log [NGF] = 2.40) neurite outgrowth at aggrecan concentrations of 37.5 µg/mL (8.103 ± 0.92 µm/cell; *p* < 0.05), 150 µg/mL (7.91 ± 0.36 µm/cell; *p* < 0.05), and 300 µg/mL (7.25 ± 0.14 µm/cell; *p* < 0.05) was significantly reduced when compared to outgrowth in the absence of bound aggrecan (11.09 ± 0.94 µm/cell) (Figure 4C). At 500 ng/mL NGF (log [NGF] = 2.70) only a concentration of 300 µg/mL aggrecan caused a significant reduction in neurite outgrowth (7.04 ± 0.09 µm/cell; *p* < 0.05) when compared to neurite outgrowth in the absence of bound aggrecan (10.53 ± µm/cell) (Figure 4D). Due to the high level of inhibition of 150 µg/mL aggrecan, below the level of saturation (greater inhibition at 300 µg/mL aggrecan), this concentration was optimal for the following studies on enzymatic digestion of aggrecan. 

**Figure 5:**
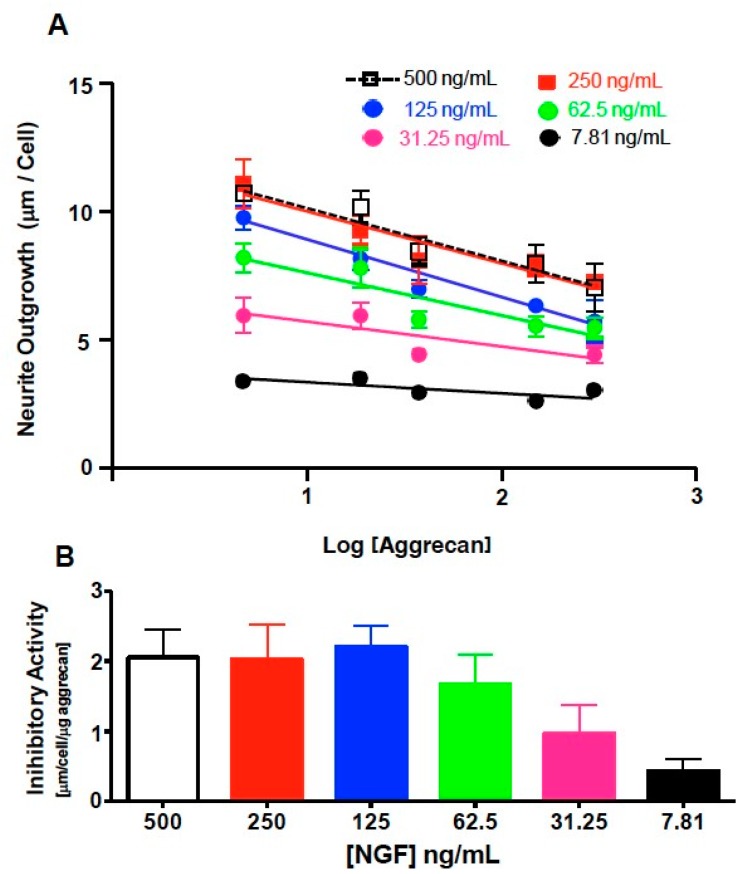
Graphical analysis of inhibitory activity. (**A**) Neurite outgrowth at 500, 250, 125, 62.5, 32.15, and 7.81 ng/mL NGF are graphed as a function of the log of the aggrecan concentration. (**B**) Bar graph plotting the inhibitory activity as a function of NGF concentration.

Since the goal of optimizing and constructing the high-throughput assay was to develop a robust, reproducible, and quantitative way to compare various proteoglycan and structural variants, we have also developed a way to calculate an “Inhibitory Activity” from the data collected (Figure 5). By constructing curves of the amount of neurite outgrowth for a given concentration of NGF against the log of aggrecan concentration, a linear relationship is observed (Figure 5A). The negative of the slope of this linear curve was defined as the inhibitory activity with units of [(µm/cell)/(log(µg/mL)aggrecan)] (Figure 5B). This slope represents the length in µm per cell that is reduced for every ten-fold increase in aggrecan concentration. Analyzing the aggrecan dose-response data in this manner shows the dependence of the inhibitory effect on NGF concentration. At lower concentrations of NGF (7.81 and 32.15), aggrecan has a low inhibitory activity (0.433 ± 0.17; 0.969 ± 0.41), while at higher concentrations of NGF (125, 250, 500 ng/mL), aggrecan has a higher, consistent, and specific inhibitory activity (2.203 ± 0.31; 2.030 ± 0.49; 2.054 ± 0.40, respectively) with an average of 2.1 [(µm/cell)/(log(µg/mL)aggrecan)] (Figure 5B). For concentrations of NGF greater than 125 ng/mL, the inhibition is aggrecan-specific, and not dependent upon NGF concentration.

### 3.4. Quantification of Inhibition of NGF-Stimulated Outgrowth Due to Aggrecan

**Figure 6:**
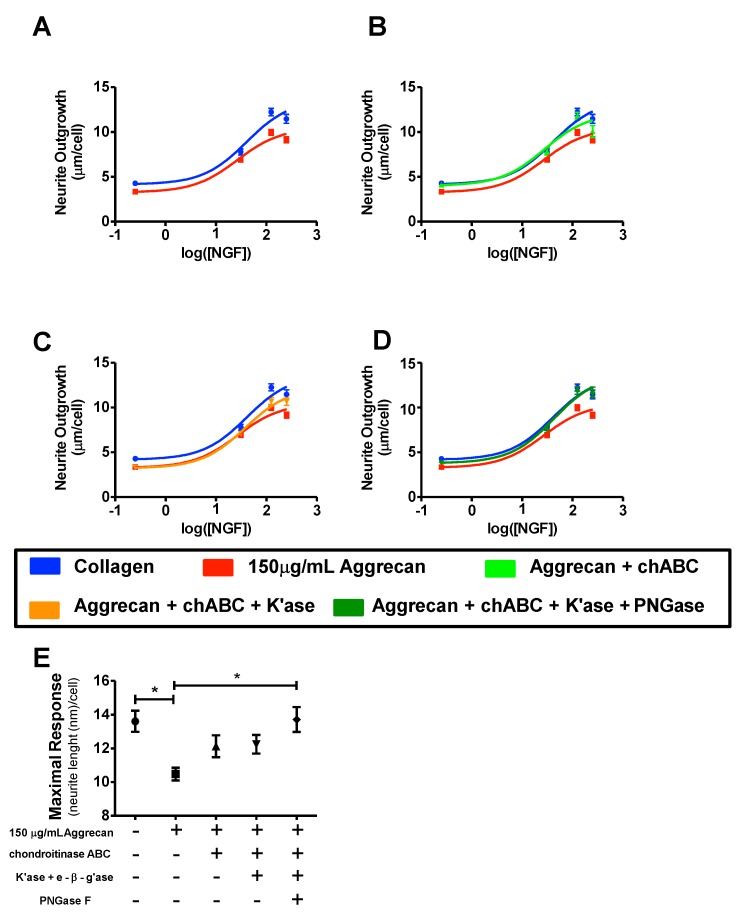
Inhibition of neurite outgrowth by enzymatically modified aggregan. (**A**–**D**) Dose response curves comparing neurite growth on collagen substrate alone and collagen plus undigested or variably digested aggrecan. The results for collagen only and collagen + undigested aggrecan are shown in each panel for comparison. (**A**) Intact aggrecan (**B**) chondroitinase ABC; (**C**) chondroitinase ABC + keratanase II/endo-β-galactosidase digestion; (**D**) chondroitinase + keratanase II/endo-β-galactosidase + PNGaseF digestion. (**E**). Statistical analysis: the first comparison (first bar) was between collagen and undigested aggrecan. The second comparison (second bar) was between intact aggrecan and variably enzyme digested aggrecan. * *p* < 0.05 following bonferonni post-hoc analysis.

In Figure 6A–D, data points are graphed on a log(dose) vs. response plot, and subsequently a sigmoidal curve was fit to those data points. Neurite outgrowth was measured in response to 0.25 ng/mL, 31.25 ng/mL, 125 ng/mL, and 250 ng/mL NGF. From this curve, one can determine the maximal response, defined as the response at the highest dose of NGF used (250 ng/mL). In the absence of aggrecan (collagen-coated surface only), NGF produces a maximal response of 13.62 ± 0.62 µm/cell, while in the presence of undigested 150 µg/mL aggrecan, this maximal response is significantly reduced to 10.48 ± 0.38 µm/cell (Figure 6A,E). Following removal of the chondroitin sulfate chains (Figure 6B,E), the maximal response is increased to a value of 12.13 ± 0.65 µm/cell. Subsequent digestion of keratan sulfate chains (Figure 6C,E) did not result in a significantly further change in the maximal response (12.25 ± 0.55 µm/cell). With removal of all three oligosaccharides (CS, KS, N-glycans) (Figure 6D,E) the maximal response is virtually identical to the collagen-only control (13.62 ± 0.62 µm/cell). Statistical analysis (Figure 6E) shows that aggrecan significantly reduces the maximal response to 250 ng/mL NGF when compared to control (no aggrecan) conditions. In addition, removal of CS, KS, and N-glycans restores the maximal response to that of collagen alone, and is significantly different when compared to intact aggrecan. The first comparison (first bar) was between collagen and undigested aggrecan. The second comparison (second bar) was between intact aggrecan and variably enzyme-digested aggrecan. These results suggest that CS does inhibit neurite outgrowth of NS-1 cells. Subsequent removal of KS does not further abolish inhibition. Further removal of N-glycans increases neurite outgrowth to control levels. 

## 4. Discussion

The high-throughput assay described here will be a useful tool to study the effect of CSPGs and other proteoglycans upon neurite outgrowth. One of the major limitations to understanding the interaction between neurite outgrowth and proteoglycans has been the absence of a purely quantitative approach to measure this interaction. This study presents a purely quantitative, robust, and simple assay that can measure the effect of substrate-bound molecules on neurite outgrowth. Quantitative assays of interactions between neurons and extracellular matrices have not been frequently employed in earlier studies, with prior reports using the outcome of growth or no growth as their leading measure [35] and often, when quantitation has been performed, data have not been presented in a concentration-response form [14]. In this report, we present a neurite outgrowth assay that allows for the quantification of small and robust differences in outgrowth length. This method represents a way in which subtle changes to PG structure and post-translational modifications can quantitatively be examined and will allow a greater understanding of the PG–neurite interaction.

Presently, there is no means of directly comparing the inhibitory potential of different proteoglycans. Our recent report [2] discussed the subtle changes in growth cone morphology in response to different structural variants of aggrecan. However, besides the apparent growth across a CSPG stripe, there was no way to determine which PG was more inhibitory than another. The field of proteoglycan effects upon neurite outgrowth has lacked a way to directly determine the differences in various PGs’ abilities to inhibit neurite outgrowth. This assay and form of analyses may be a simple and robust way to directly compare different PGs and variants thereof to each other.

One limitation of our approach is that only NS-1 cells were used in these experiments. NS-1 cells are a subclone of the PC12 cell line and are NGF-responsive pheochromocytoma cells [36]. Being an immortalized cell line, they may not be the most physiologically relevant representation of neurites in the injured spinal cord or brain. It would be useful to verify and validate this assay using primary neurons from various parts of the central nervous system. Further, isolation of different types of neurons (i.e., serotonergic vs. glutamatergic) and use of this assay will allow investigators to decipher whether different neuronal types interact differentially with PGs.

CS has been widely reported to be the major inhibitory component of CSPGs. In fact, the enzyme used in this project to remove CS chains (chABC) has shown promise as a therapeutic intervention to promote neuroregeneration in the injured spinal cord. For instance, treatment with chABC following animal models of spinal cord injury enhances functional recovery, axonal sprouting, and axonal re-growth [25,37,38,39,40]. Our study was consistent with these earlier investigations, and we have shown that removal of CS chains using chABC allowed for greater neurite outgrowth. This is in agreement with a number of other analyses in vitro that used various cell types and neuronal lineages [3,19,41,42,43].

Often overlooked as a contributor to the biological activity of PGs, in particular aggrecan, are keratan sulfate chains, although some labs have focused on this GAG [44]. Prior experiments show that KS has opposing effects on neurite outgrowth compared to CS, dependent on cell type. For instance, CS is permissive to dopaminergic neurons of the striatum while KS is inhibitory using ventral mesencephalic explants in culture [45]. In addition, during development in the rat there is a mosaic distribution of CS and KS in the striatum with CS localization in areas of dopaminergic axon tracts and KS abundant in areas lacking dopaminergic innervation [46]. This suggests that depending on the cell type, CS and KS may have differential effects. In the work described here, we have not seen evidence for KS-mediated inhibition of neuritogenesis.

**Figure 7:**
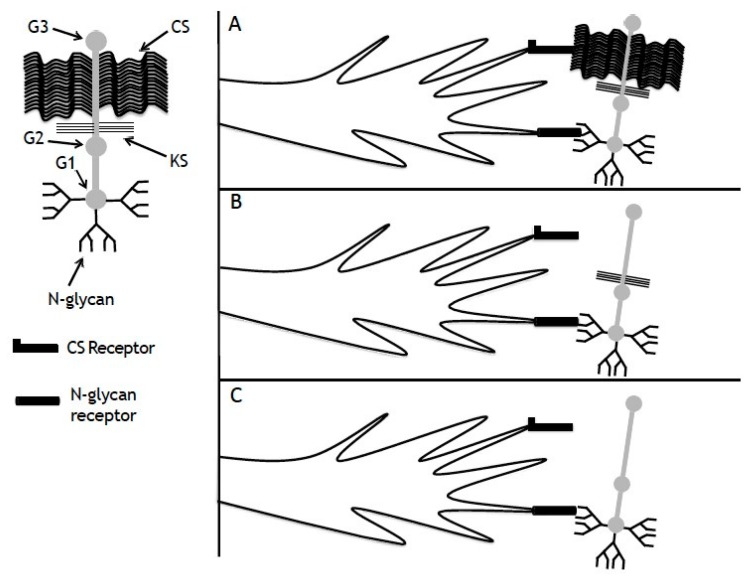
Effect of aggrecan post-translational modifications on neurite outgrowth. **(A**) In the presence of intact aggrecan there is reduced maximal growth when compared to growth on collagen, likely due to the activation of CS receptors and receptors for N-glycans. (**B**) Removal of CS abolishes some inhibition, but there is no apparent effect of further removal of KS (**C**), suggesting no KS-receptor interaction. Removal of N-glycans following CS and KS removal abolishes inhibition, suggesting an interaction of N-glycans with a receptor on the growth cone.

Aggrecan depleted in CS and KS, but containing only N-glycans, is inhibitory to neurite outgrowth, but following PNGase-F digestion, is permissive. Historically, N-glycans are the least-investigated post-translational modifications often associated with proteoglycans. N-glycans are highly branched sugar chains and are found mostly on the G1-domain of aggrecan. N-glycans are associated with many receptors and structural proteins and are important for cellular responses [47,48,49]. There are only a limited number of publications discussing the role of N-glycans in neurite outgrowth. PC12 cells that were transfected with β1,4 N-acetylglucosaminyl transferase III (enzyme that catalyzes formation of bisecting GlcNAc in N-glycans) display reduced neurite outgrowth. In addition, increasing the presence of N-glycans through transfection upregulates expression of EGF and integrins [50]. The cytoplasmic protein amphoterin (a heparan sulfate PG) is localized to leading edges in spreading and motile cells (i.e., growth cone) and is bound by the receptor for advanced glycation end products (RAGE) through N-glycans attached to the receptor. Binding of amphoterin by this receptor promotes neurite outgrowth [51,52]. Further, transfection of PC12 cells with N-acetylglucosaminyltransferase (enzyme necessary for production of N-glycans) enhances β1 integrin-ECM interactions and leads to an increase in neurite outgrowth on laminin and collagen [53]. More relevant to our study, the adhesive molecule NCAM binds to oligomannosidic carbohydrates (i.e., N-glycans) and this interaction is important for NCAM-laminin-1 association. Further, neurite outgrowth of early postnatal mouse cerebellar neurons is reduced in the presence of oligomannosidic carbohydrates [54]. It is therefore quite possible that N-glycans, following CS and KS digestion, inhibit neurite outgrowth through association with adhesion molecules, blocking their interaction with other proteins that may promote neurite outgrowth. Therefore, removal of N-glycans would allow for greater outgrowth through less interference with adhesion molecules, as we observed. A model illustrating the interactions of aggrecan with growth cone receptors is shown in Figure 7. In the presence of fully glycosylated aggrecan there is reduced maximal growth when compared to growth on collagen alone (Figure 7A), likely due to the activation of CS receptors and receptors for N-glycans. CS removal abolishes some inhibition (Figure 7B), but there is no apparent effect of further removal of KS (Figure 7C) suggesting that there is no KS-receptor interaction. Removal of N-glycans following CS and KS digestion abolishes inhibition, suggesting an interaction of N-glycans with a receptor on the growth cone. 

Post-translational modifications of PGs may have important clinical relevance. The enzymatic digestion of aggrecan performed in this study revealed important effects caused by the post-translational modifications associated with PGs. Preclinically, the removal of CS from the injured spinal cord is successful at improving neuronal regeneration and functional outcome [55]. However, in these models neurons are not necessarily regrowing to reform the damaged connections, and both functional recovery and regrowth is limited. It is likely that other factors of the damaged spinal cord, including other GAGs, are playing key roles in recovery. KS is known to play an important role in synaptic plasticity [56], and as shown here, the removal of CS appears to remove the inhibition of neurite outgrowth, likely through the removal of CS, but also greater access to KS. In rodent models of spinal cord injury, there is an increase in keratan sulfate immunoreactivity associated with the injury site along with macrophages, reactive microglia, and oligodendrocyte progenitors [27,57]. Whether or not the KS is restricting neuronal regrowth and plasticity is up for debate, as studies suggest conflicting conclusions. For instance, in the developing chick CNS, retinal axons grow along a path of keratan sulfate proteoglycans [58] suggesting a growth-promoting or permissive effect, while a specific developmentally regulated keratan sulfate PG (claustrin) inhibits cell adhesion and neurite outgrowth of embryonic optic lobe neurons [44]. The interaction of keratan sulfate proteoglycans with outgrowing neurites needs to be understood to a greater depth to fully realize the beneficial or deleterious effects of removing CS chains from PGs. In our study, the enzymatic removal of N-glycans from aggrecan allowed neurite outgrowth to rebound to control levels. The effect we observe from PNGase F digestion of aggrecan containing N-glycans alone suggests that enzymatic removal of N-glycans may enhance neurite outgrowth in the glial scar. Further studies are needed to determine if a similar effect could be achieved in vivo.

## 5. Conclusions

We report the use of a high-throughput assay to determine the effect of various post-translational modifications of the CSPG aggrecan upon neurite outgrowth from NS-1 cells. We have shown in this study that this assay represents a powerful method to investigate multiple components of the CSPG-neurite interaction. Using a sequential digestion approach, we found that chondroitin sulfate and N-glycans, but not keratan sulfate, contribute to inhibition of neurite outgrowth by substrate-bound aggrecan. For the first time, we have shown that N-linked oligosaccharides on aggrecan contribute to its inhibition of neuritogenesis. A better understanding of the role of CSPGs in neurite outgrowth will further medicine’s ability to repair damage to the CNS and rebuild complex neural circuitry.
